# Identification of RNAi hypoallergic bread wheat lines for wheat-dependent exercise-induced anaphylaxis patients

**DOI:** 10.3389/fnut.2023.1319888

**Published:** 2024-01-16

**Authors:** María H. Guzmán-López, Violeta Ruipérez, Miriam Marín-Sanz, Isabel Ojeda-Fernández, Pedro Ojeda-Fernández, José Antonio Garrote-Adrados, Eduardo Arranz-Sanz, Francisco Barro

**Affiliations:** ^1^Functional Genomics Laboratory, Plant Breeding Department, Institute for Sustainable Agriculture, Spanish National Research Council (IAS-CSIC), Córdoba, Spain; ^2^College of Agricultural Engineering, University of Valladolid, Palencia, Spain; ^3^Clínica Ojeda, Clínica de Asma y Alergia Doctores Ojeda, Madrid, Spain; ^4^Excellence Unit, Institute of Biology and Molecular Genetics, University of Valladolid—Spanish National Research Council (CSIC), Valladolid, Spain

**Keywords:** RNAi, gluten, IgE, wheat, wheat-dependent exercise-induced anaphylaxis (WDEIA), reverse-phase high-performance liquid chromatography (RP-HPLC), non-gluten proteins (NGPs)

## Abstract

Wheat-dependent exercise-induced anaphylaxis (WDEIA) is one of the most severe forms of wheat allergy. It occurs in patients when they exercise after ingesting wheat-containing foods. Nowadays, the only possible alternative for WDEIA patients is to avoid such foods. This study investigated the potential of six RNA of interference (RNAi) wheat lines with low-prolamin content as alternatives for WDEIA patients. For that purpose, a high performance-liquid chromatography (HPLC) analysis was performed to evaluate differences in gluten protein fractions among these lines. Next, western blots were conducted to measure the immunoglobulin E (IgE) reactivity to wheat proteins in sera from five WDEIA patients. Additionally, monoclonal antibodies (moAb) recognition sites and the IgE binding sites were searched in all peptides identified by LC-MS/MS after protein digestion. The results showed a 61.4%–81.2% reduction in the gliadin content of the RNAi lines, accompanied by an increase in their high-molecular weight (HMW) glutenin content compared to the wild type bread wheat line (WT). In all cases, the reduction in gliadin content correlated with a decrease in IgE reactivity observed in the sera of WDEIA patients, highlighting the E82 and H320 lines. These two RNAi lines exhibited a ≤90% reduction in IgE reactivity. This reduction could be attributed to an absence of IgE binding sites associated with α- and ω5-gliadins, which were present in the WT. Overall, these lines offer a potential alternative for foodstuff for individuals with WDEIA.

## 1 Introduction

Wheat is one of the most widely grown cereals in the world, mainly due to the distinct viscoelastic properties of its dough. Wheat flour contains a complex mixture of gluten proteins, directly related to bread-making quality, and non-gluten proteins (NGPs), metabolic and structural functions playing a secondary role in wheat quality (1). Meanwhile, gluten proteins comprise about 80% of the total flour protein and include two different protein groups: gliadins and glutenins. The monomeric gliadins can be further classified into α-/β-, γ-, and ω-gliadins according to their mobility in acid-PAGE (A-PAGE). Additionally, ω-gliadins can be divided into ω1,2-gliadins and ω5-gliadins based on their N-terminus sequences; beginning with ARE/KELQS and SRLL, respectively (2). The polymeric glutenins consist of high-molecular-weight glutenin subunits (HMW-GSs) linked by disulfide bonds to low-molecular-weight glutenin subunits (LMW-GSs) (3).

Gluten proteins are also responsible for a wide range of immune responses related to human pathologies such as celiac disease (CD), a chronic enteropathy linked to the human leukocyte antigens (HLA) HLA-DQ2 and HLA-DQ8 in susceptible individuals accounting for a 1% of the worldwide population (4, 5). Another pathology associated with gluten is non-celiac wheat sensitivity (NCWS), characterized by intestinal and extra-intestinal symptoms after consuming wheat-based products (6, 7). In contrast to CD, NCWS does not elicit an autoimmune response. Other pathologies related to gluten include allergies such as baker’s asthma and wheat-dependent exercise-induced anaphylaxis (WDEIA). Both wheat allergies are triggered by a range of wheat proteins that stimulate immunoglobulin E (IgE) production, which leads to the activation of mast cells and basophils by cross-linking.

WDEIA is one of the most severe forms of wheat allergy and occurs in patients when they engage in physical exercise after the ingestion of wheat-containing foods (8). Other co-hypothesized factors are drugs, alcohol, or stress that may influence the allergic response by increasing gastrointestinal permeability or tissue transglutaminase activation (2, 9). This pathology may present a range of clinical symptoms, including urticaria, angioedema, generalized erythema, and wheezing, and can progress to life-threatening anaphylactic shock (10, 11). Because of its symptoms, WDEIA frequently goes undiagnosed and is mistaken for other pathologies such as chronic urticaria, exercise-induced or idiopathic anaphylaxis (12). The prevalence of this pathology has been reported to be between 0.10%–0.80% in adults regardless of ethnicity and region (13–19).

Diverse IgE-binding sites (epitopes) have been associated with WDEIA (2, 20–25). The major allergens responsible for this pathology are the ω5-gliadins. In fact, some authors prefer to use the term “ω5-gliadin allergy” instead of WDEIA (26, 27). The primary epitope for the ω5-gliadins has been identified as QQX1PX2QQ, where X1 can be L, F, S, or I, and X2 can be Q, E, or G, as determined through cDNA cloning (21, 24, 28, 29). A study by Matsuo et al. (21) found that 80% of WDEIA patients strongly reacted to the ω5-gliadins, while the remaining patients reacted to the HMW glutenins. Thus, HMW glutenins are also considered major allergens, being QQPGQ, QQPGQGQQ, and QQSGQGQ major epitopes for WDEIA (21, 24). Other minor epitopes have been described in α/β-gliadins, γ-gliadins, ω1,2-gliadins, and LMW-GSs (26, 29, 30).

Various alternatives have been developed to address the issue of major allergens present in wheat-based foods (2). One of these approaches involves altering allergen epitopes by enzymatic degradation, deamidation, or the addition of reducing agents. Another approach involves the use of wheat lines with reduced allergenicity. These lines can either be obtained through classic plant breeding methods or through genetic engineering techniques like RNAi. Diverse hypoallergenic wheat lines lacking ω5-gliadins have shown promising results in reducing reactivity to serum IgE in wheat-allergic patients while maintaining flour characteristics. However, none of these lines are still safe for WDEIA patients to consume.

Consequently, a lifelong gluten-free diet is the only possible treatment for wheat-related pathologies. However, both gluten and wheat are used extensively in the food industry, which makes avoiding these products considerably challenging. This leads to transgressions in the diet which can be up to 60% (31). Therefore, reducing or eliminating specific gluten proteins constitutes a desirable aim for selecting less immunogenic varieties while preserving wheat organoleptic qualities.

RNAi technology has been successfully used for the down-regulation and knock-out of gliadin genes in bread and durum wheat, resulting in wheat lines with low gluten content and low stimulatory capacity of triggering CD and NCWS (32–34). The present study aimed to evaluate the IgE-mediated reactivity of protein extracts from the flour of six RNAi wheat lines with low-prolamin by using WDEIA patients’ sera. To that, we quantified the gliadin and glutenin content to select the RNAi lines and carried out total protein digestions. Later, we compared the reactivity mediated by IgE of control and WDEIA patients’ sera to RNAi wheat flours. Finally, we carried out a proteomic analysis of these lines to further characterize peptide composition and IgE recognition sites.

## 2 Materials and methods

### 2.1 Plant material

Six RNAi lines and one control line were used in this study. The control line, denoted as BW208, is a bread wheat line from the International Maize and Wheat Improvement Center (CIMMYT), and the six RNAi lines (E33, E82, E83, D793, D894, and H320) were obtained from this line and are further described in Table 1 (32–36). Briefly, E33, E82, and E83 lines were transformed with two plasmids for the silencing of the ɣ-gliadins and the α- and ω-gliadins; both hairpin RNA (hpRNA) silencing fragments under the control of a D-hordein promoter (33). D793 and D894 lines were transformed with one plasmid for silencing the α- and ω-gliadins under the control of a ɣ-gliadin promoter (35). H320 line was transformed with two plasmids, one targeting the α-gliadins and the other targeting the ω-gliadins, both under the control of a D-hordein promoter (36).

**Table 1:** Description of bread wheat lines used in this study, including plasmids and RNAi fragments used.

	Plasmid 1		Plasmid 2			
Lines	Name	RNAi fragment	Name	RNAi fragment	Promoter	References
BW208	NA	NA	NA	NA	NA	NA
E33	pDhpg8.1	g8.1	pDhp_ω/α	α + ω	D-hordein	(34)
E82	pDhpg8.1	g8.1	pDhp_ω/α	α + ω	D-hordein	(34)
E83	pDhpg8.1	g8.1	pDhp_ω/α	α + ω	D-hordein	(34)
D793	pGhp_ω/α	α + ω	NA	NA	γ-gliadin	(36)
D894	pGhp_ω/α	α + ω	NA	NA	γ-gliadin	(36)
H320	pDhp_α/βZR	α/β ZR	pDhp_ω4ZR	ω4	D-hordein	(37)

### 2.2 Patient sera

Human sera were obtained from eight adults: three healthy adults (1 female, 2 males, mean age: 44, mean IgE against ω5-gliadin: < 0.35 kU/L) and five patients with a confirmed clinical history of anaphylaxis episodes and WDEIA positive diagnosis (1 female, 4 males, mean age: 48, mean IgE against ω5-gliadin: 25.51 kU/L; Supplementary Table S1). The diagnosis was confirmed through (i) cereal flour prick-tests and (ii) hybridizing sera with bread wheat grain proteins and later determination of reactivity mediated by IgE. Interviews were conducted to ascertain that no healthy participant had experienced an allergic episode after consuming wheat products. Sera were collected from the participating individuals and stored at −80°C until needed. All experiments involving human sera were carried out in accordance with the principles embodied in the Declaration of Helsinki of 1965 (as revised in Brazil 2013). This study uses samples obtained from a by-product of routine care or industry. Therefore, the study did not require to be reviewed or approved by an ethics committee. Patients also provided informed consent for their use in scientific research.

### 2.3 Gliadins and glutenins quantification by reverse-phase (RP)-HPLC

The gliadin and glutenin fractions were extracted from wheat flour and quantified by RP-HPLC as reported in Marín-Sanz et al. (37). Briefly, 100 mg of flour were homogenized with 3 × 670 μL of 60% (v/v) ethanol and, incubated at RT for 10 min while continuously shaking. The samples were then centrifuged, and the supernatants containing the gliadin fraction were collected. The remaining pellet served as the raw material for extracting glutenins. As a result, each sample received 3 × 670 μL of C1 buffer, which contained 50% (v/v) 1-propanol, 2 M urea, 0.05 M Tris-HCl (pH 7.5), and 2% (w/v) dithiothreitol (DTT). Samples were vortexed and then incubated at 60°C for 30 min. Following centrifugation, the supernatants containing the glutenin fraction were collected. The three collected supernatants were mixed and filtered in both cases through a 0.45 μm nylon filter.

The gliadins and glutenins were quantified from each sample using a 1200 Series Quaternary LC System liquid chromatography from Agilent Technologies (Santa Clara, United States) coupled to a DAD UV-V detector. Ten microliters of protein extracts were injected into a LiChrospher^®^ 100 RP8 column (Merck, Darmstadt, Germany). The integration procedure was performed automatically by Agilent Technologies ChemStation software with minor adjustments. Three replicate analyses were carried out on separate extracts for each RNAi line and control.

### 2.4 Total proteins and non-gluten proteins determination

The total protein content of whole flour was determined using the Kjeldahl nitrogen content (%*N* × 5.7) according to the standard ICC method No. 105/2 (38). The NGPs, expressed in percentage of dried weight (% DW), were calculated for each line as follows: Total protein in % − (Prolamin content in μg/mg × 10)/(100 − Moisture in %), where prolamin content is the sum of the gliadins and glutenins.

### 2.5 Wheat protein extracts and IgE immunoblotting analyses

For immunoblotting, wheat grain proteins were extracted as described in Gil-Humanes et al. (39). Briefly, 25 μL of a protein extraction buffer [62.5 mM Tris-HCl, pH 6.8, 2% (w/v) sodium dodecyl sulfate (SDS), 1.5% (w/v) DTT, 10% (v/v) glycerol and 0.002% bromophenol blue] per mg of flour was added to each sample. After a 30 min incubation period at 70°C, the samples were centrifuged at 14,000 g for 2 min, and the supernatant was collected.

The reactivity mediated by IgE of bread wheat proteins was determined through western blotting. Total protein extracts were separated through 12% SDS-PAGE and transferred to a 0.45 μm polyvinylidene difluoride (PVDF) membrane (Thermo Fisher Scientific, Waltham, United States). Once transferred, the membrane was incubated for 1 h in a TBS blocking solution (50 mM Tris-HCl, 150 mM NaCl, pH 7.5) with 5% goat serum. 1:10 dilutions of human sera samples were incubated for 20 h at 4°C. After washing three times, the membrane was incubated for 1 h with a 1:5,000 dilution of a human anti-IgE secondary antibody conjugated to peroxidase (Invitrogen Life Technologies, Carlsbad, United States). Additionally, the gliadin fraction of RNAi and WT lines were extracted following the method described in Gil-Humanes et al. (40). Afterward, proteins were separated through 10% SDS-PAGE and later subjected to a western blot using the same protocol as above. In this latter case, an anti-gliadin antibody (Sigma-Aldrich, Burlington, United States) was used. Regardless of the antibody and extract used, proteins were detected by a Pierce ECL system (Thermo Fisher Scientific, Waltham, United States) and quantified by band gel densitometry using Quantity One (Bio-Rad). Experiments were carried out in triplicate.

### 2.6 Protein digestion and proteomic data analysis

Flour from control and RNAi wheat lines was subjected to protein extraction, digestion, and proteomic analysis as described by Haro et al. (41). Firstly, UPEX solution and 60% ethanol were used to extract gluten protein. After extraction, each sample’s total protein content was precipitated using the methanol/chloroform method. Forty micrograms of protein pellets were resuspended, denatured, and reduced for digestion. Digestions of each sample were performed by adding chymotrypsin endoproteinase MS Grade (Thermo Fisher Scientific, Waltham, United States) or sequence grade-modified trypsin (Sigma-Aldrich, Burlington, United States) in a 1/20 ratio (w/w). The samples were then incubated at 37°C overnight on a shaker and then dried out by evaporation.

Digested peptides were then subjected to 1D-nano LC ESI-MS/MS analysis using a nano liquid chromatography system coupled to a high-speed Triple TOF 5600 mass spectrometer with a Nanospray III Source as previously described in Vaquero et al. (7). Raw data files were converted to Mascot generic files (.mgf), then searched against *Triticum* spp. NCBI database using the Mascot Server v. 2.5.0 (Matrix Science, London, United Kingdom). The search parameters and additional details were described in Vaquero et al. (7). Finally, custom Python scripts were used to search for monoclonal antibody recognition sites and IgE binding sites in unique peptides with perfect match or one mismatch.^1^ The IgE-binding epitope sequences were obtained from Matsuo et al. (30) for the gluten proteins, non-specific lipid transfer protein 1, serpins, and α-purothionins. These scripts were also used to sort these unique peptides into the different types of gluten proteins: ω-, α- and γ-gliadins, HMW-, and LMW-GSs. The sequences searched in the unique peptide database are described in Supplementary Table S2 (28, 42–45).

## 3 Results

### 3.1 Protein profiles of RNAi lines

**Figure 1:**
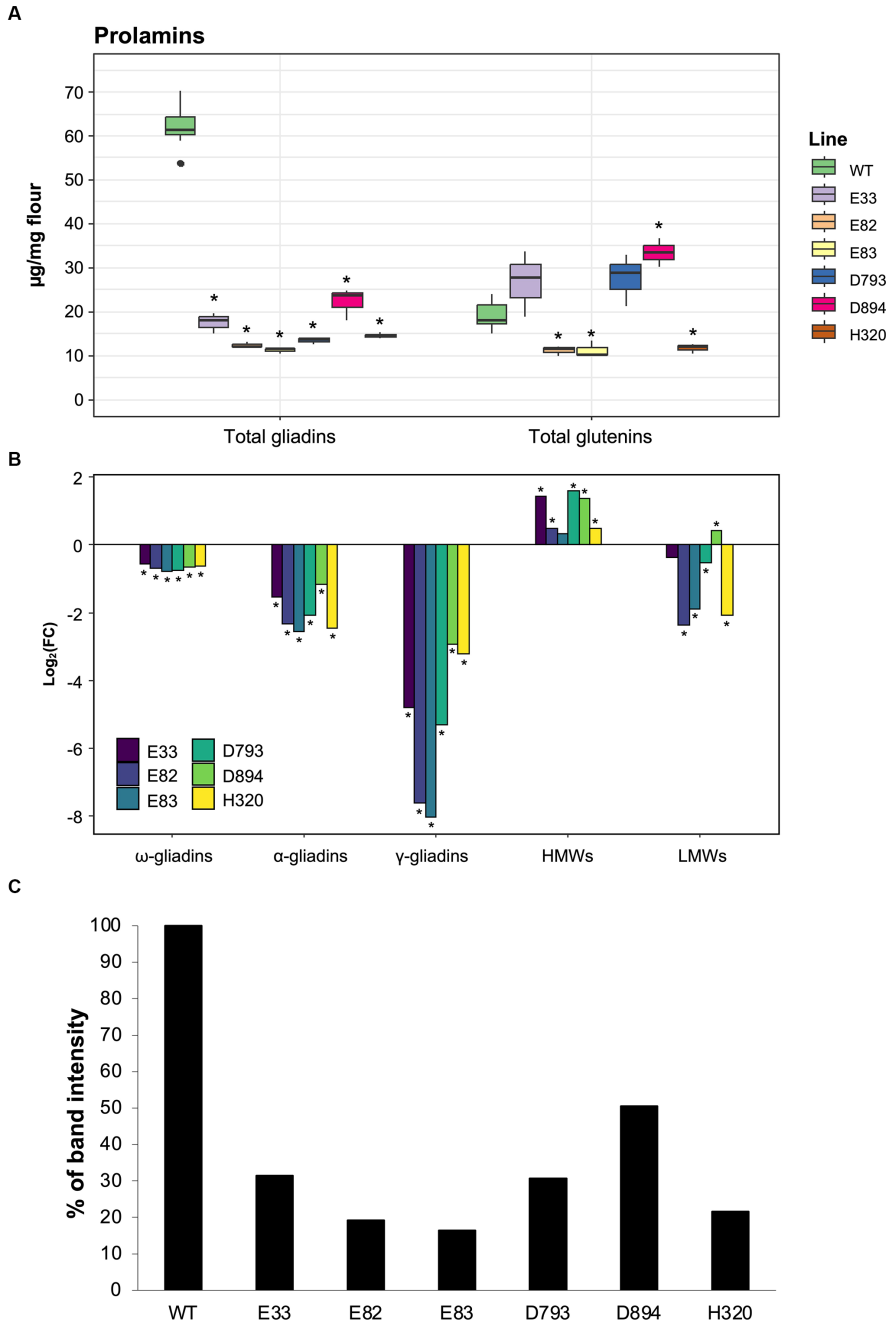
**(A)** Total content of gliadins and glutenins in each line quantified by RP-HPLC analysis. **(B)** The log_2_(FC) of each of the gliadin and glutenin fractions was quantified by RP-HPLC analysis for each line compared to the WT (BW208). Mann–Whitney tests were performed for comparison each line and the WT. ^*^p < 0.05. **(C)** Percentage of gliadin band intensity in SDS-PAGE of RNAi lines compared to the WT. WT, wild type bread wheat line.

First, RP-HPLC analysis was performed to characterize the differences between the RNAi and control lines for the gluten protein fractions, gliadins and glutenins (Figure 1). RNAi lines harbor different hpRNA fragments with gliadins as their common target. Furthermore, the hpRNAs are driven by different endosperm-specific promoters. Hence, differences in the content of the gluten protein fractions are likely to result from the efficiency of the promoters and their match in silencing their respective targets. As shown, gliadins were significantly reduced by 61.4%–81.2% in the RNAi lines compared to the WT (Figure 1A). Additionally, the total gliadin content was quantified by SDS-PAGE and band gel densitometry in all lines (Figure 1C). These data also showed a 44.9%–96.2% reduction of gliadins in all RNAi lines. Regardless of the type of protein quantification, the E82 and E83 lines presented the lowest gliadin content. Interestingly, according to the RP-HPLC data, the total glutenin levels increased by 85.7% in the D894 line, while the E82, E83, and H320 lines presented a 33.5%–43.0% reduction compared to the WT. No significant differences were found in the glutenin content between the D793, E33, and WT lines. Figure 1B shows that all three gliadin fractions were decreased in the RNAi lines: with the γ-gliadins exhibiting the largest silencing of all gliadin fractions, particularly in the E82 and E83 lines. In contrast, the HMW-GSs were increased in all RNAi lines, while the LMW-GSs decreased. The only exception was the D894 RNAi line, which presented an increase in both glutenin fractions compared to the BW208 WT line. Overall, the strong decrease in gliadins leads to an increase in the HMW-GSs and is also compensated by the NGPs; the greater the decrease, the greater the compensation, so that the total protein content remains around 13% in all RNAi lines (Supplementary Figure S1).

### 3.2 IgE reactivity to total protein extracts

**Figure 2:**
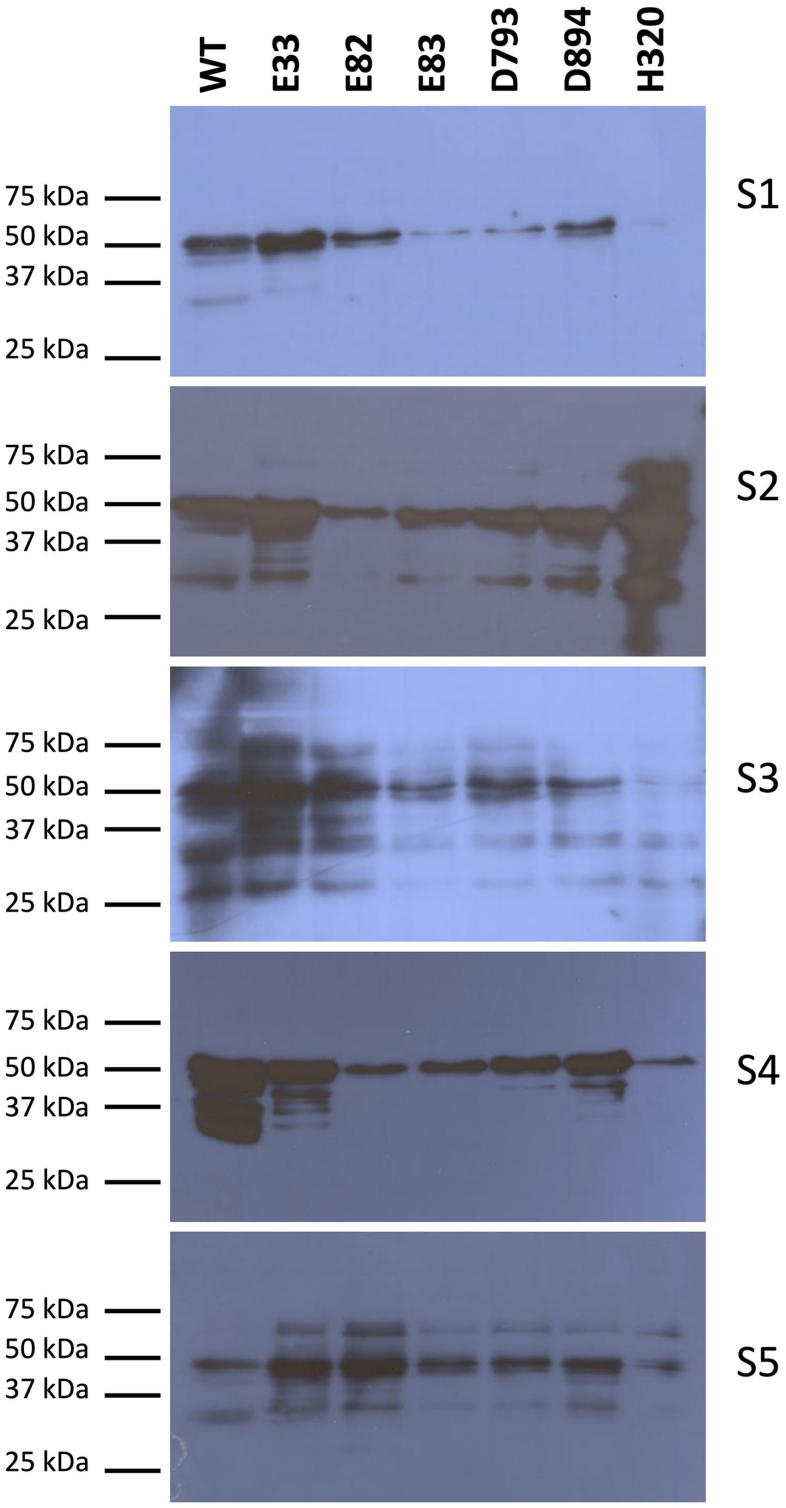
IgE specific immunodetection in total protein extracts of WT and RNAi bread wheat lines in each WDEIA patient (S1-5). WT, wild type bread wheat line.

**Figure 3:**
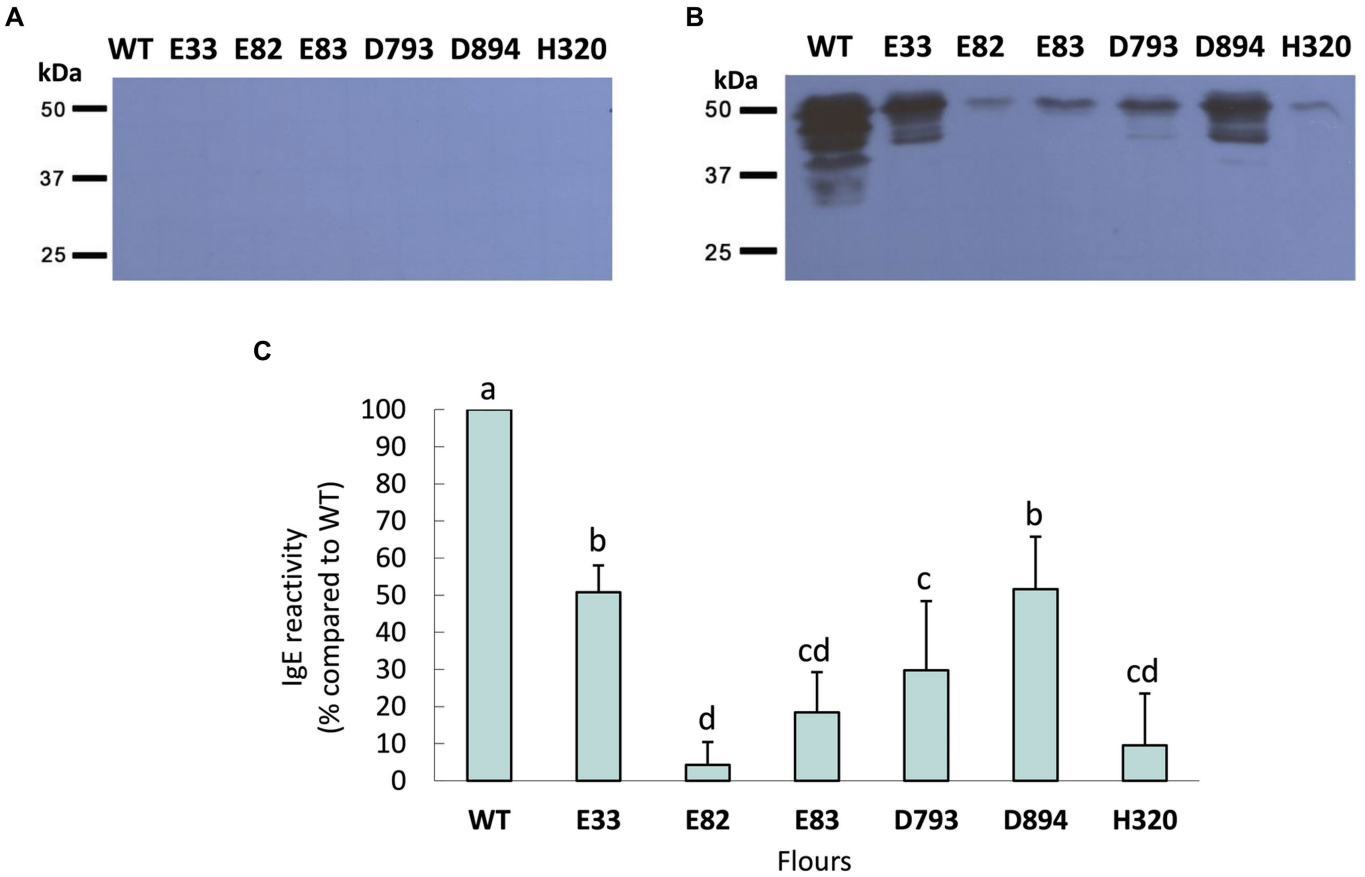
Reactivity mediated by IgE against WT and RNAi bread wheat flours in a sera mix of **(A)** control patients and **(B)** WDEIA patients. **(C)** The reactivity is presented as a percentage of reactivity when compared to the WT (100% reactivity). Data correspond to the average ± SD of three different experiments. Completely randomized *p*-values were obtained by ANOVA. Means followed by a common letter are not significantly different by the LSD test at the 5% of significance. *p* < 0.001. WT, wild type bread wheat line.

Next, western blots were carried out to quantify the reactivity mediated by IgE of protein extracts from the WT and the RNAi lines. Initially, the serum from each WDEIA patient and healthy adult was incubated individually with total protein extracts from each low-gliadin RNAi and control lines (Figure 2; Supplementary Figure S2). When the serum of each WDEIA patient was used individually for the assay, E82, E83, and H320 lines presented the highest reduction in reactivity across all patients when compared to the WT. Additionally, a sera mix composed of proportional parts of each patient’s serum was also incubated with total protein extracts from each WT and RNAi line. The results obtained are represented in Figure 3. These results indicate that all RNAi lines presented a significant decrease in the reactivity mediated by IgE when compared to the WT (Figures 3B,C). These data concord with the total content of gliadins in the flours, as these lines present a high silencing in the expression and accumulation of gliadins. Thus, the reduction in gliadin content implies a decrease in allergenicity in wheat, highlighting E82 and H320 lines, which showed a marked reduction in reactivity (≤90% compared to the WT) in patients with WDEIA. No reactivity was observed in serum from healthy individuals incubated with the protein extracts (Figure 3A).

### 3.3 Proteomic data analysis from RNAi and control bread wheat flours

**Figure 4:**
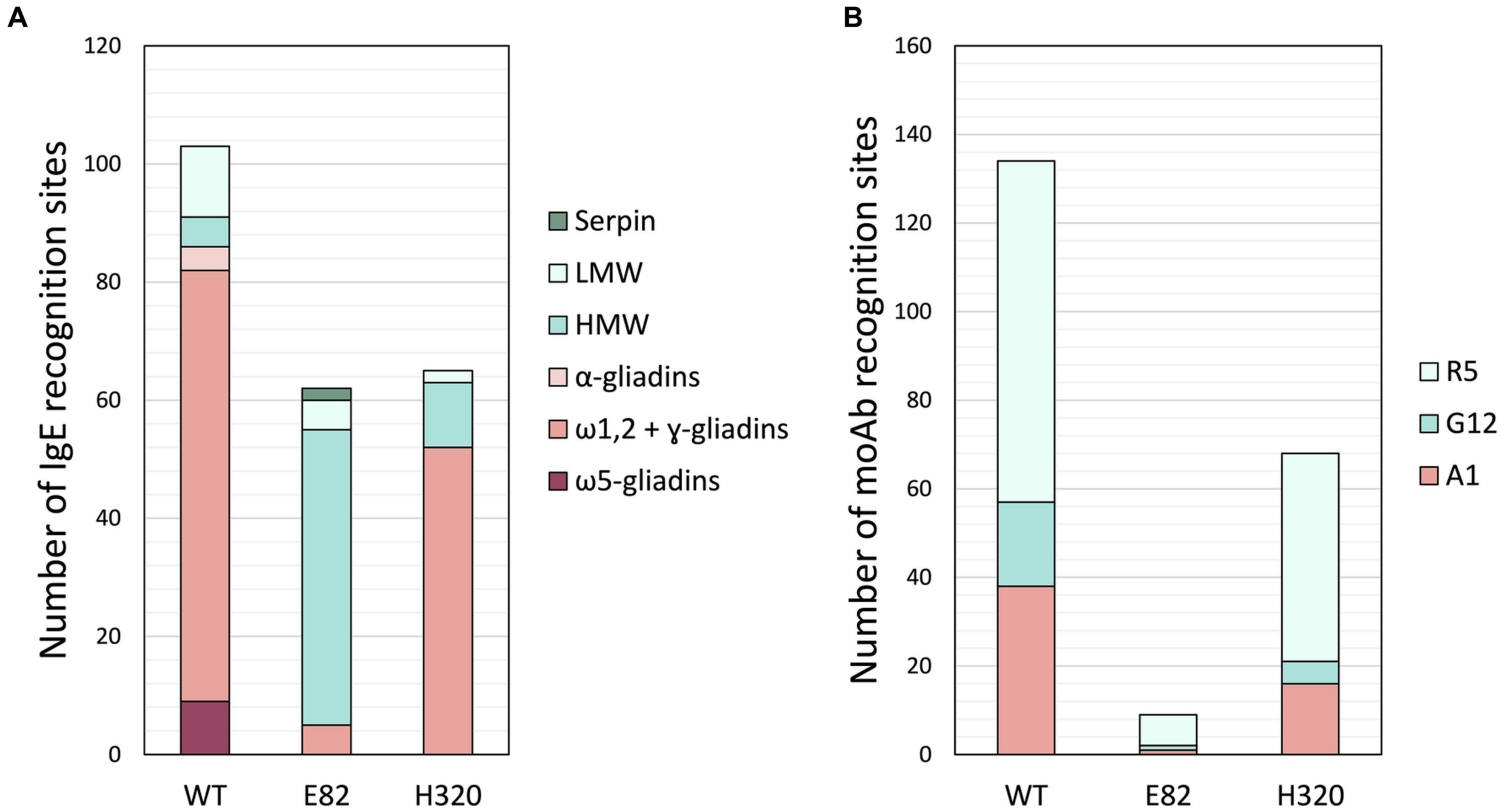
Number of hits of **(A)** IgE and **(B)** moAb recognition sites in the unique peptides of BW208 (WT), E82, and H320 lines. WT, wild type bread wheat line.

As H320 and E82 lines exhibited the lowest IgE reactivity of WDEIA patients’ sera, a proteomic analysis of their flour was carried out to characterize them further. Protein extraction of flour from bread wheat RNAi and WT lines was performed and digested with chymotrypsin or trypsin. Then, peptides were identified by LC-MS/MS analysis, in which custom Phyton scripts searched for monoclonal antibodies (moAb) recognition sites and the IgE binding sites in all peptides identified by LC-MS/MS after protein digestion. On the one hand, the IgE binding sites have been previously linked to various illnesses, including WDEIA, baker’s asthma, and wheat allergy (28, 30). On the other hand, R5, G12, and A1 are the most popular moAbs utilized to determine the presence of gluten peptides. The ELISA R5 method is recommended by the Working Group on Prolamin Analysis and Toxicity (WGPAT) for determining gluten and is described as a Type I analysis method by the Codex Alimentarius (CODEX STAN 118-1979). The G12 and A1 moAbs were designed to recognize the most immunogenic gluten peptide of α-gliadins, the 33-mer (45, 46). The results obtained are displayed in Figure 4. When compared to the WT, both RNAi lines exhibited fewer moAbs recognition sites and IgE binding sites. The E82 and H320 lines presented a 90%–97% and 38%–57% reduction of the moAbs recognition sites in comparison to that of the WT, respectively (Figure 4B). Regarding the IgE binding sites, neither the ω5-gliadin nor the α-gliadin epitopes were present in the peptides from the RNAi lines. However, the E82 and H320 did present 6.8 and 71.2% of ω1,2- and ɣ-gliadin epitopes of the WT, respectively. As for the IgE binding sites related to glutenins, both lines presented fewer LMW binding sites but more HMW than the WT (Figure 4A). The E82 and H320 exhibited 41.7% and 16.7% of the LMW binding sites present in the WT. However, the E82 and H320 lines presented more HMW binding sites than the control line, 10 and 2.2 times more HMW, respectively. Interestingly, only two IgE binding hits were found in serpins in the E82 line.

## 4 Discussion

In this study, we compared the allergenicity of six RNAi lines (E33, E82, E83, D793, D894, and H320) in sera from WDEIA patients (33, 35, 47). All the bread wheat lines were transformed for the silencing of gliadins under either a D-hordein or γ-gliadin promoter. The ω- and α-gliadins were targeted in all lines, and in E33, E82, and E83 lines, the ɣ-gliadins were also down-regulated.

Initially, we characterized the gliadin and glutenin content of all RNAi lines through RP-HPLC analysis. Our results showed a significant reduction in the total gliadin content of these lines compared to the control line, with the γ-gliadins exhibiting the largest silencing of all fractions. The highest level of gliadin silencing was observed in the E82 line; 81.2%. Notably, only the D894 line showed a significant increase in its total glutenin fraction compared to the control, while the E82, E83, and H320 lines showed a significant reduction in this protein fraction. This reduction in the total glutenin fraction in most lines was due to a large decrease in the LMW glutenins, while HMW glutenins increased with respect to the control. Additionally, the total protein content remained comparable for all lines. Similar compensatory effects were observed in bread wheat lines in which α-gliadin genes were edited through CRISPR/Cas9 technology (48). Furthermore, these effects have also been observed in other RNAi lines designed to target gluten proteins (49, 50). For instance, certain RNAi lines targeting the ω5 gliadin showed an increase in NGPs, glutenin subunits, and α-gliadins (50). In contrast, when ω1,2- gliadins were silenced, compensation occurred with HMW-GSs and NGPs (49). Furthermore, these protein compensations have also been reported in bread and durum wheat lines in which the α-, γ- and ω-gliadins were down-regulated by RNAi (39). In this latter study, these adjustments in the protein expression of these RNAi lines had significant consequences on the characteristics of the flour as greater amounts of HMW glutenins are generally linked with stronger flours.

Western blots were carried out with WDEIA patients’ sera to determine the IgE reactivity to total protein extracts. When the serum from each WDEIA patient was incubated separately with total protein extracts, the E82, E83, and H320 showed the lowest IgE reactivities compared to the WT. These results correlate with the changes observed in the content of total gliadin and glutenin fractions in these lines.

However, the response of each patient to every protein extract varied widely. Patient S2, for instance, exhibited the highest reactivity to the H320 protein extract among IgE present in the serum, whereas it was one of the least reactive extracts for the rest of the patients. Interestingly, the same S2 patient displayed the lowest reactivity to E82, which was also one of the least reactive lines in S1 and S4 patients. This variation of IgE reactivity in patients with WDEIA has been observed previously in other studies. Altenbach et al. (8) found variations in IgE reactivity between patients to the same protein extracts, which could be attributed to variations in IgE levels among patients or other underlying food allergies and conditions that these patients may have. In the case of LMW glutenins, this latter study noted that amounts of IgE reactivity to specific LMW glutenins were not related to protein abundances, suggesting that there are differences in the affinity of sera for specific types of LMW glutenins. Skoczowski et al. (51) also observed that the immunological response of WDEIA patients to wheat proteins is highly specific and dependent on the wheat variety and serum applied.

To mitigate inter-patient differences, we incubated a serum mix comprising equal proportions of each patient’s serum with total protein extracts from WT and RNAi lines. The IgE-mediated reactivity significantly decreased in all RNAi lines compared to the WT. Notably, E82 and H320 lines demonstrated a considerable reduction in IgE reactivity in patients with WDEIA: ≤90% relative to the WT. While the IgE binding profiles are highly specific, some immunodetected proteins are probably significant allergens as they are shared by all the patients analyzed. Based on the strength of the immunodetection by individual sera, the proteins of around 50 kDa exhibited the highest immunoreactivity. Skoczowski et al. (51) also reported the immunodetection of 49 kDa NGP in sera from WDEIA patients, as well as LMW glutenins and gliadins of similar size.

To learn about which protein fraction could be related to causing an IgE reaction in each patient, we performed a proteomic analysis of our less immunoreactive RNAi lines—E82 and H320— to characterize them further. Digested peptides of flour from RNAi and WT lines were identified by LC-MS/MS analysis. This peptide composition served as input for custom Phyton scripts that searched for monoclonal antibodies (moAb) recognition sites and IgE binding sites previously described (28, 30). Overall, both E82 and H320 lines presented the fewest moAb recognition sites and IgE binding sites when compared to the WT, which concord with the previously observed decrease in both the IgE reactivity and gliadin fraction content.

Both RNAi lines presented none of the IgE binding sites searched present in α- and ω5-gliadins. These results are consistent with the lower immunoreactivity observed in the sera of WDEIA. Given that ω5-gliadin is regarded as the most critical epitope for WDEIA, these results explain the reduced IgE reactivity in the sera of WDEIA patients (21, 22, 28). In fact, several studies have demonstrated that ω5-gliadin is a promising candidate for diagnosing immediate wheat allergy and not only WDEIA (20, 28, 29, 52). Furthermore, both lines presented a reduction in LMW-GSs epitopes as well as in ω1,2- and ɣ-gliadin binding epitopes compared to the WT. However, it is worth noting that the IgE binding sites present in these lines exhibited distinct characteristics. Notably, the H320 line displayed a higher abundance of ω1,2- and ɣ-gliadin binding sites than the E82 line. Conversely, the E82 line presented a greater amount of HMW-GSs epitopes in comparison to the H320. Despite these differences, both lines showed minimal reactivity, suggesting that none of these epitopes are likely responsible for the IgE reactivity observed in the patients’ sera.

Although both RNAi lines presented exhibited fewer IgE binding epitopes in LMW-GSs than the WT, they presented more HMW-GSs epitopes. Nevertheless, the immunoreactivity assays with patients’ sera did not show IgE reactivity with HMW glutenins. Previous research has recognized two groups of patients with WDEIA based on the IgE reactivity to wheat proteins: one group reacts to ω5-gliadin (which accounts for 80% of patients), while the other group reacts to HMW-GSs (21, 22). Additionally, sensitization to ω5-gliadin and HMW-GSs have been reported to be different depending on the age of the patient; higher sensitization rates of ω5-gliadin are found in over 20 years-old patients, while higher sensitization rates of HMW-GSs is found in younger patients (53). Consequently, the reduced IgE reactivity observed in patients’ sera could be attributed to the fact that these patients, all above 20 years old, might belong to the more common subset, namely the ω5-gliadin-reacting group.

Regarding the moAb recognition sites, both lines presented fewer R5, G12, and A1 recognition sites than the WT. Nowadays, the ELISA R5 method is the most widely used to determine gluten presence in wheat-derived products. While R5 moAb has been designed to recognize peptides of rye secalins, the G12 and A1 moAb recognize the 33-mer, the main immunogenic gluten peptide of α-gliadins for CD patients (54). Thus, the reduced presence of these moAb recognition sites supports the idea that the E82 and H320 lines also have lower immunogenic capacity. In fact, the E82 line has already been tested in a pilot study with CD patients, and it did not elicit an immune response after a short-term oral challenge (55). Hence, these findings could benefit individuals suffering from various wheat-related pathologies.

Based on our findings, we can conclude that all RNAi lines analyzed in this study exhibited reduced gliadin content, which was accompanied by an increase in their HMW-GSs content in comparison to the WT. In all cases, the reduction in gliadin content correlated with a decrease in IgE reactivity observed in the sera of WDEIA patients, although immunoblotting patterns were specific to both patients and wheat genotypes. Additionally, our observations suggest that the reduced IgE reactivity could be mainly attributed to the absence of IgE binding sites found in α- and ω5-gliadins. Overall, these lines may offer a potential alternative for consumption by individuals with WDEIA. Nonetheless, to draw a firm conclusion about the practicality of these lines, it would be necessary to perform an oral provocation test in a larger group of WDEIA patients, utilizing both wild type and our RNAi lines.

## Data availability statement

The raw data supporting the conclusions of this article will be made available by the authors, without undue reservation.

## Ethics statement

Ethical approval was not required for the studies involving humans because this study uses samples obtained from a by-product of routine care or industry. Therefore, the study did not require to be reviewed or approved by an ethics committee. Patients also provided informed consent for their use in scientific research. The studies were conducted in accordance with the local legislation and institutional requirements. The participants provided their written informed consent to participate in this study.

## Author contributions

MG-L: Data curation, Formal analysis, Investigation, Writing – original draft, Writing – review & editing. VR: Formal analysis, Investigation, Methodology, Writing – review & editing. MM-S: Data curation, Formal analysis, Methodology, Writing – review & editing, Investigation. IO-F: Investigation, Resources, Writing – review & editing. PO-F: Investigation, Methodology, Resources, Writing – review & editing. JG-A: Formal analysis, Investigation, Methodology, Writing – review & editing. EA-S: Conceptualization, Investigation, Resources, Supervision, Writing – review & editing. FB: Conceptualization, Formal analysis, Funding acquisition, Investigation, Methodology, Project administration, Resources, Supervision, Visualization, Writing – review & editing.

## Footnotes

^1^ https://github.com/MiriamMarinS/ProtEp.git
