# Supplementary material for: Identification of RNAi hypoallergic bread wheat lines for wheat-dependent exercise-induced anaphylaxis patients
Source: Front Nutr. 2024 Jan 16;10:1319888. doi: 10.3389/fnut.2023.1319888 (PMC10824911; doi:10.3389/fnut.2023.1319888)
Supplement: Supplementary file 1 [file Data_Sheet_1.docx]

Supplementary Material

# Supplementary Figures and Tables

## Supplementary Figures


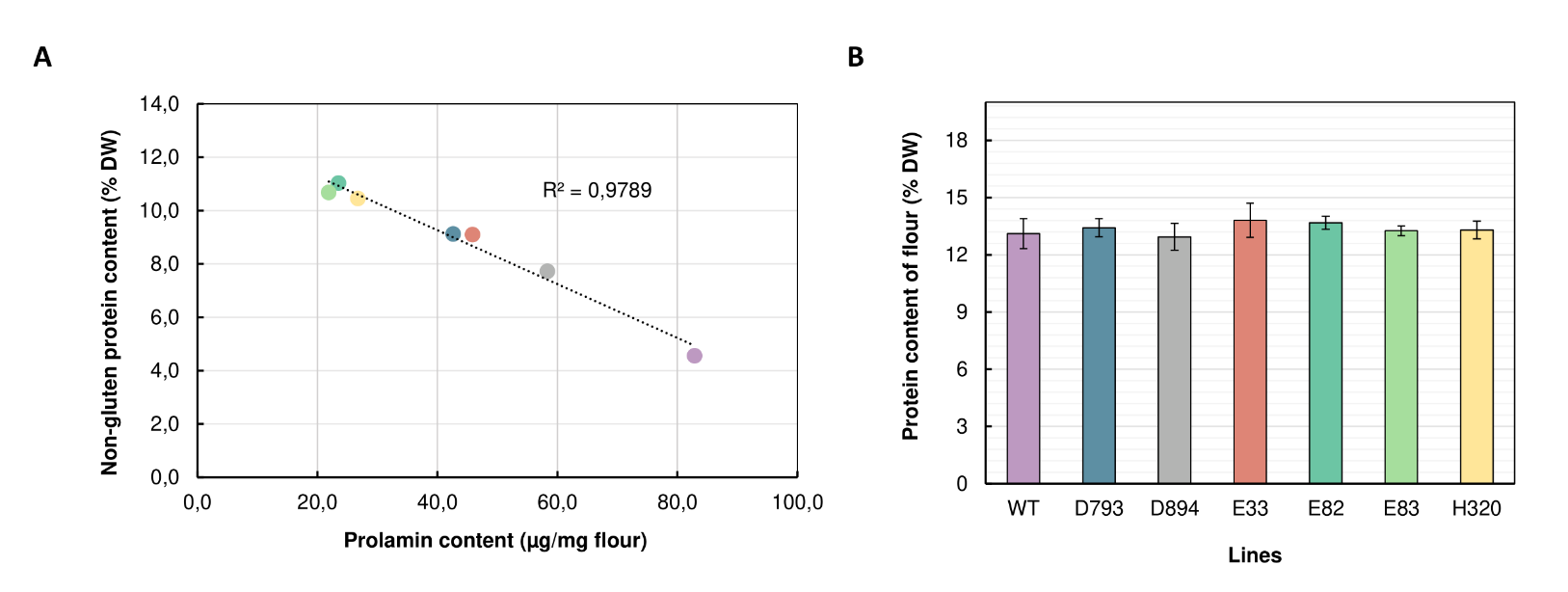


**Supplementary Figure 1.** **(A)** Linear correlation between non-gluten protein content and prolamin content in each line. **(B)** Protein content of flour in each RNAi line. DW: dry weight, WT: wild type bread wheat line.


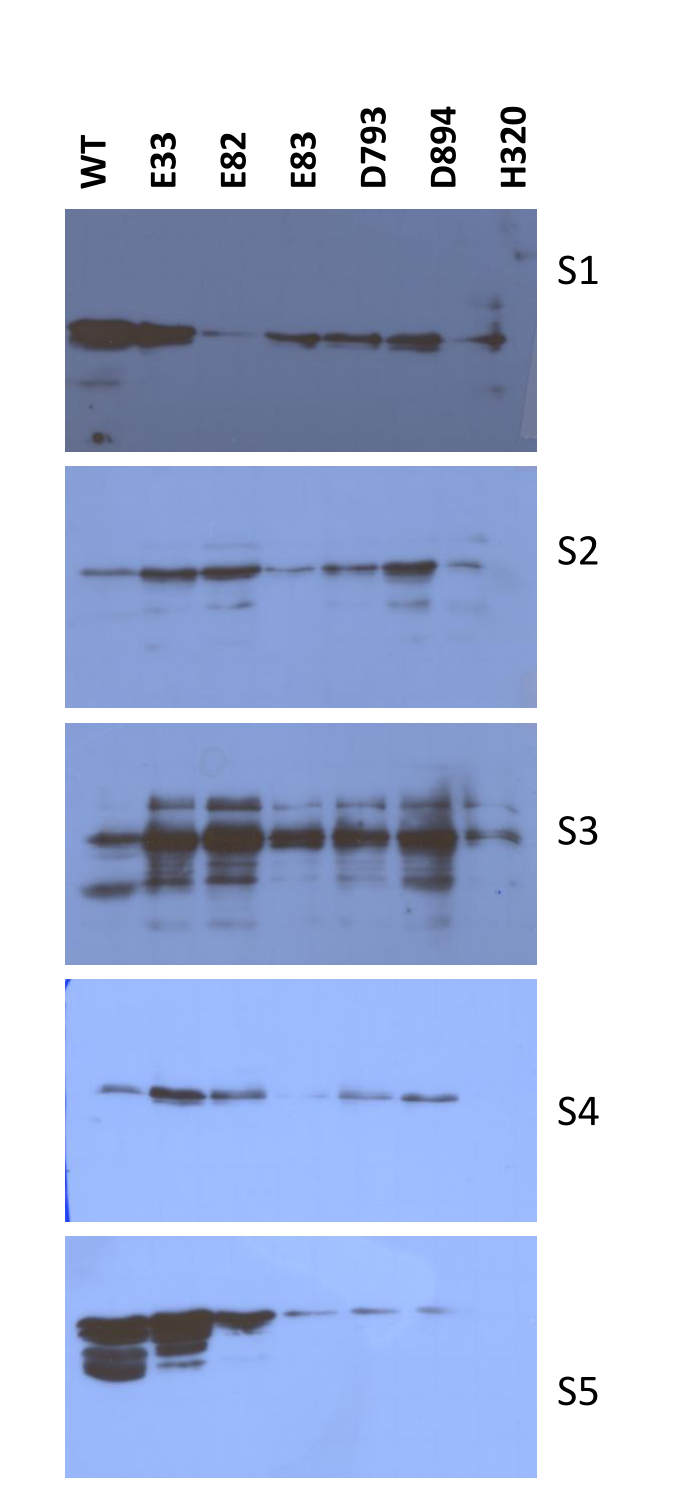


**Supplementary Figure 2.** Results of IgE specific immunodetection against gliadin proteins in the wild type and RNAi bread wheat lines in each WDEIA patient (S1-S5) of repetition nº2. WT: wild type bread wheat line.

## Supplementary Tables

**Supplementary Table 1.** Human sera description including serum code, origin (healthy adult or WDEIA patient), age and IgE against ω5-gliadin (kU/L). WDEIA: Wheat-dependent, exercise-induced anaphylaxis. IgE: Immunoglobulin E.

| **Serum code** | **Origin** | **Sex** | **Age** | **IgE against ω-5-gliadin (kU/L)** | |
| --- | --- | --- | --- | --- | --- |
| C1 | Healthy adult | female | 27 | < 0,35 |  |
| C2 | Healthy adult | male | 56 | < 0,35 |  |
| C3 | Healthy adult | male | 50 | < 0,35 |  |
| P1 | WDEIA patient | male | 73 | 9,73 |  |
| P2 | WDEIA patient | female | 39 | 10,8 |  |
| P3 | WDEIA patient | male | 55 | 10,3 |  |
| P4 | WDEIA patient | male | 41 | 7,81 |  |
| P5 | WDEIA patient | male | 32 | 88,9 |  |

**Supplementary Table 2.** Epitope names and sequences of moAb recognition sites and IgE binding sites. References in which each one is described are also listed in the table. moAb: monoclonal antibody.

| **Type** | **Epitope name** | **Peptide sequence** | **Ref** |
| --- | --- | --- | --- |
| **moAb recognition sites** | G12_1 | QPQLPY | [47] |
|  | G12_2 | QPQLPF |  |
|  | G12_3 | QPQLPL |  |
|  | G12_4 | QPQQPY |  |
|  | A1_1 | QLPFPQP | [47] |
|  | A1_2 | QQPFPQP |  |
|  | A1_3 | QLPYPQP |  |
|  | A1_4 | QQPYPQP |  |
|  | A1_5 | QQPYPQE |  |
|  | R5_1 | QQPFP | [46] |
|  | R5_2 | QQQFP |  |
|  | R5_3 | LQPFP |  |
|  | R5_4 | QLPFP |  |
| **IgE-binding sites** | IgE_ω5_1 | QQIPQQQ | [30] |
|  | IgE_ω5_2 | QQFPQQQ |  |
|  | IgE_ω5_3 | QQSPEQQ |  |
|  | IgE_ω5_4 | QQSPQQQ |  |
|  | IgE_ω5_5 | QQLPQQQ |  |
|  | IgE_ω5_6 | QQYPQQQ |  |
|  | IgE_ω5_7 | PYPP |  |
|  | IgE_ω5_8 | QQFHQQQ |  |
|  | IgE_ω5_9 | QSPEQQQ |  |
|  | IgE_ω5_10 | YQQYPQQ |  |
|  | IgE_ω5_11 | QQPPQQ |  |
|  | IgE_ω5_12 | QQQLPQQQ |  |
|  | IgE_ω5_13 | QQQFPQQQ |  |
|  | IgE_HMW_1 | QQPGQ |  |
|  | IgE_HMW_2 | QQPGQGQQ |  |
|  | IgE_HMW_3 | QQSGQGQ |  |
|  | IgE_α_1 | VRVPVPQLQP |  |
|  | IgE_α_2 | QEQVPLVQQQ |  |
|  | IgE_α_3 | VQQQQFPGQQ |  |
|  | IgE_α_4 | QQQFPGQQQQ |  |
|  | IgE_α_5 | YLQLQPFPQP |  |
|  | IgE_α_6 | QILQQILQQQ |  |
|  | IgE_α_7 | LQIPEQSQCQ |  |
|  | IgE_α_8 | QEQKQQLQQQ |  |
|  | IgE_α_9 | SFQQPQQQYP |  |
|  | IgE_α_10 | LALQTLPAMC |  |
|  | IgE_α_11 | YIPPHCSTTI |  |
|  | IgE_γ_1 | QPQQPFPQ |  |
|  | IgE_ω12_1 | QPQQPFPQ |  |
|  | IgE_LMW_1 | HQQQPIQQQP |  |
|  | IgE_LMW_2 | QQPIQQQPQQ |  |
|  | IgE_LMW_3 | QQFPQQQPCS |  |
|  | IgE_LMW_4 | PFVHPSILQQ |  |
|  | IgE_LMW_5 | QCSPVAMPQS |  |
|  | IgE_LMW_6 | LPQIPQQSRY |  |
|  | IgE_LMW_7 | QSRYEAIRAI |  |
|  | IgE_LMW_8 | QQQPP |  |
